# How Well Do Seniors Estimate Distance to Food? The Accuracy of Older Adults’ Reported Proximity to Local Grocery Stores

**DOI:** 10.3390/geriatrics4010011

**Published:** 2019-01-10

**Authors:** Benjamin W. Chrisinger, Abby C. King, Jenna Hua, Brian E. Saelens, Lawrence D. Frank, Terry L. Conway, Kelli L. Cain, James F. Sallis

**Affiliations:** 1 Stanford Prevention Research Center, Stanford University School of Medicine, 1070 Arastradero Road, Suite 300, Palo Alto, CA 94304, USA; king@stanford.edu (A.C.K.); jhua1224@stanford.edu (J.H.); 2 Department of Health Research & Policy (Epidemiology), Stanford University School of Medicine, Stanford, CA 94305, USA; 3 Department of Pediatrics, Seattle Children’s Research Institute and University of Washington, Seattle, WA 98121, USA; brian.saelens@seattlechildrens.org; 4 School of Community and Regional Planning, University of British Columbia, Vancouver, BC V6T 1Z2, Canada; ldfrank@ud4h.com; 5 Department of Family & Preventive Medicine, University of California, La Jolla, CA 92093, USA; tlconway@ucsd.edu (T.L.C.); kcain@ucsd.edu (K.L.C.); jsallis@ucsd.edu (J.F.S.)

**Keywords:** built environment, older adults, food environment, food access, walkability, perception, GIS, neighborhood environment walkability scale

## Abstract

(1) Background: Findings from observational studies of relations between neighborhood environments and health outcomes underscore the importance of both objective and perceived experiences of those environments. A clearer understanding of the factors associated with discrepancies between these two assessment approaches is needed to tailor public health interventions to specific populations. This study examined how individual and neighborhood characteristics affect perceptions of supermarket distance, particularly when perceptions do not match objective measures. (2) Methods: Participants were older adults (*n* = 880) participating in the Senior Neighborhood Quality of Life Study in the Seattle/King County, WA or Baltimore/Washington, DC regions. Two main analyses were conducted. The primary outcome for Analysis I was participants’ geographic information systems (GIS)-based objective network distance to the closest supermarket. Generalized linear mixed models with block group-level random effects were used to assess associations between objective supermarket distance and individual/neighborhood characteristics. The primary outcome for Analysis II was a categorical “accuracy” variable, based on participants’ perceived distance to the nearest supermarket/grocery store relative to the objective distance, assuming a walking speed of 1.0 m/s. Multivariate log-linear models fit neural networks were used to assess influential covariates. (3) Results: Several significant associations with objective distance to the nearest supermarket were observed, including a negative relationship with body mass index (BMI) (95% CI = −45.56, −0.23), having walked to the supermarket in the last 30 days (−174.86, −59.42), living in a high-walkability neighborhood, and residing in Seattle/King County (−707.69, −353.22). In terms of participants’ distance accuracy, 29% were classified as accurate, 33.9% were “Underestimators”, 24.0% “Overestimators”, and 13.2% responded “Don’t Know”. Compared to Accurate participants, Overestimators were significantly less likely to have walked to the supermarket in the last 30 days, and lived objectively closer to a supermarket; Underestimators perceived significantly higher pedestrian safety and lived objectively further from a supermarket; and Don’t Know were more likely to be women, older, not living independently, and not having recently walked to the supermarket. (4) Conclusions: Both modifiable and nonmodifiable factors influence the accuracy of older adults’ perceptions of their proximity to the nearest supermarket. Recent experience in walking to the closest supermarket, along with personal safety, represent potentially modifiable perceived environmental factors that were related to older adults’ accuracy of perceptions of their neighborhood food environment.

## 1. Introduction

Built environment characteristics, such as walkability, safety, and food environment, are understood to affect the adoption and maintenance of diet and physical activity behaviors affecting a wide range of chronic disease outcomes [1,2]. Importantly, these relationships appear to endure across the life course. As individuals age, the built environment around where they live supports or deters their ability to maintain healthy behaviors known to help prevent or reduce frailty and other aging-related morbidities [3,4,5,6]. 

Critical to our interpretation of how the built environment affects the health of older adults are the objective built environments where individuals live, work, and play, as well as the attitudes and perceptions individuals hold about these places. Perceived neighborhood environmental factors have been found to be associated with walking in older adult populations [5]. Individuals’ perceptions of safety and other walkability domains are of particular importance to physical activity outcomes [7,8,9]. For instance, Lee et al. found that perceived but not objective social and environmental variables were significantly related to neighborhood satisfaction among a large study of adults in two metropolitan regions in the United States [10]. Meanwhile, a systematic review of neighborhood effects on physical activity among older adults by Yen and colleagues found “fairly consistent” evidence for the associations between both objective and perceived environments and physical activity, but noted that these constructs are likely to be linked to health outcomes in different ways [4]. With this in mind, questions arise for neighborhood-based public health research and practice concerning the accuracy of perceptions of neighborhood environmental domains that could support greater physical activity or the use of health-promoting resources and amenities or, alternatively, present barriers to health-promoting activities through misperception or unawareness of local environmental factors.

One domain of particular interest is the neighborhood food environment. Several studies have found that individuals’ perceived healthy food access is significantly associated with objective physical distance to supermarket retailers [11,12]. One study by Barnes et al. found that living further from the nearest supermarket was associated with a reduction in perceived healthy food access [12]. Other studies have investigated whether food environment perceptions are related to diet [13], including whether they mediate possible dietary changes related to improved healthy food access [14]. Among older adults in a rural setting, Sharkey and colleagues found that both objective (e.g., living closer to a supermarket or produce retailers) and perceived food access (e.g., perceived number of grocery retailers nearby, fruit/vegetable variety availability) were related to a higher intake of fresh and processed fruits and vegetables [15]. Caspi et al. found that among residents of low-income housing, perceptions of healthy food access were significantly related to fruit and vegetable consumption, though objective distance to supermarkets was not [13]. Furthermore, participants who lived within a kilometer of a supermarket but did not report a supermarket to be within walking distance of home ate significantly fewer fruits and vegetables than those who lived similarly close and reported living close to such outlets [13]. 

Systematic reviews have also reported that the food environment—including the availability of or access to retailers that carry healthy food items [16]—may be a potential moderator of fruit and vegetable consumption for older adults [17]. However, contradictory findings do exist, where no relationship has been found between the perceived food environment and fruit and vegetable intake among older adults; instead, other factors, such as mobility and self-rated health, were determined to be significantly related to such intake [18]. Studies have also identified potential barriers to food shopping for older adults, such as difficulties in carrying groceries or finding items that fit their budgets and preferences [14,19], as well as perceived proximity, route characteristics from home to food retailers [5,20], and self-rated health [21,22]. Thus, even in communities with objectively high walkability, optimal food environments for older adults may have different characteristics than those for the general population [23]. 

Among seniors, the gap between built environment perception and reality has been noted in at least one study of older adults’ use of and estimated distance to neighborhood resources [24]. Here, the researcher found that public transit use was significantly related to both objective and perceived built environment variables, as well as individual characteristics. Importantly, transit use appeared to increase participants’ perceived distance to the nearest transit stop or station (e.g., seniors who were transit users overestimated how far they lived from a transit stop, while non-users underestimated it) [24]. Given the potential adverse effects or lost opportunities resulting from inaccurate neighborhood perceptions, as well as the possibly exacerbating effects of limited mobility among older adults, a better understanding of what informs and characterizes the accuracy or inaccuracy of older adults’ neighborhood perceptions is warranted.

### Study Objectives

A combination of objective and perceived data was used to answer two questions about food environment perceptions among older adults participating in the observational Senior Neighborhood Quality of Life Study (SNQLS): (1) Which individual sociodemographic and built environment characteristics were associated with objective proximity to a particular food environment resource?; and (2) Which individual sociodemographic and built environment characteristics predicted the degree to which an individual accurately perceived the distance to this resource? In this study, as in previous research, supermarkets were assessed as the primary point of food access given their size, recognizability, and year-round provisioning of fresh fruits and vegetables [12,13,15]. 

## 2. Materials and Methods

### 2.1. Study Overview

Participants in the observational multi-site senior neighborhood quality of life study (SNQLS, *n* = 883) were ages 66 years and older, able to walk at least 10 ft with or without assistive devices, able to complete study surveys in English, and lived in either the Seattle/King County, WA region or the Baltimore/Washington, DC region of the US. [25]. Between 2005 and 2008, SNQLS investigators purposively sampled from census tracts in Seattle/King County, WA and Baltimore/Washington, DC that would allow comparisons across different levels of neighborhood income and walkability. A neighborhood “quadrant” categorical variable was generated to balance participant recruitment across this research design: Low-walkability, low-income; low-walkability, high-income; high-walkability, low-income; and high-walkability, high-income. Similarly, a categorical variable for site (Seattle/King County, WA or Baltimore/Washington, DC) was also generated. Participant recruitment/consent and SNQLS methods are described in detail elsewhere [25,26].

### 2.2. Measures

#### 2.2.1. Survey Measures

Individual neighborhood perceptions were assessed by selected items from the abbreviated Neighborhood Environment Walkability Survey (NEWS-A) [27], a validated and abbreviated version of the original 98-question NEWS instrument [28,29], and included domains of aesthetics, traffic safety, infrastructure for walking and bicycling, personal safety, and pedestrian safety [27]. For each of these domains, participants assessed their neighborhood by responding to statements (e.g., “There are trees along the streets in my neighborhood”) on a 4-point Likert scale from “Strongly Disagree” to “Strongly Agree”. Participants also reported the walking time to the nearest supermarket by selecting one of six response categories: 1–5 min, 6–10 min, 11–20 min, 21–30 min, more than 30 min, or “Don’t Know”. 

Derived from participant surveys described in King et al. [25], dichotomous variables were created to control for potentially influential variables: Self-reported gender (female or male), race/ethnicity (non-Hispanic white or not), use of a cane/walker (yes or no), having finished at least some higher education (yes or no), dog ownership (yes or no), holding a valid driver’s license (yes or no), living independently (i.e., not residing in a group facility, yes or no), and having walked to the perceived nearest supermarket in the past 30 days (yes or no). Continuous variables included participant age, body mass index (BMI), household size, length of residence at current address, number of vehicles available at home, and objective network distance to the nearest supermarket. Ordinal variables included comfort walking (10-point Likert, with 1 = no confidence and 10 = complete confidence) [30], and NEWS-A component scores of aesthetics, pedestrian safety, personal safety, traffic safety, and walking/cycling facilities [27]. 

#### 2.2.2. Geographic Information Systems (GIS) Measures

Geographic information systems (GIS) were used to calculate objective network distance from participants’ home addresses to a variety of neighborhood business locations contained in a proprietary Dun & Bradstreet, Inc./Hoovers database. Four-digit standard industrial classification (SIC) codes were used to group destinations, including grocery stores and supermarkets. 

### 2.3. Accuracy of Distance to Supermarket Perception 

A categorical variable for the accuracy of perceived distance to the nearest supermarket was created in three steps. First, we estimated the time required to walk the actual network distances to the nearest supermarket for each participant, assuming a 1.0 m/s walking pace for older adults [31]. For example, a participant who lived 1.0 km from the nearest supermarket would have been assigned an estimated walk time of 16.7 min. This estimated walk time was then used to assign an objective distance category corresponding to the NEWS-A survey: 1–5 min, 6–10 min, 11–20 min, 21–30 min, and greater than 30 min. Finally, estimated walk times were compared to perceived walk times to assign participants one of three classifications: “Accurate” if participants’ estimated walk times fell within their selected perceived walk time category, “Overestimate” if their perceived walk time was greater than their estimated walk time, and “Underestimate” if their perceived walk time was less than their estimated walk time. A separate category was assigned to participants who selected “Don’t Know” for their perceived supermarket distance in the NEWS-A. To evaluate how the results might change based on assumptions of walking paces other than the 1.0 m/s pace, sensitivity analyses were conducted by constructing distance accuracy variables with slightly faster (1.2 m/s) and slower (0.8 m/s) assumed walking paces, using the same procedure as described in the Materials and Methods section. A second sensitivity analysis was also performed using a reduced dataset only including participants without any of three potential mobility constraints that could substantially slow a participant’s walking speed (use of a cane or walker, low comfort walking four blocks (self-reported score of <6 on a 10-point scale, low to high comfort), and not living independently).

### 2.4. Statistical Procedures

Continuous and ordinal variables were centered and scaled with the caret package in R, which subtracts a variable’s mean from each of its values and divides by the standard deviation [32,33]. Descriptive statistics were generated to summarize participant and neighborhood characteristics both by perceived closeness to a supermarket and accuracy of this perception. For Analysis I (variables related to objective distance to the nearest supermarket), a series of linear mixed models were generated (using the lmer function of the lme4 package) in R (Version 3.5.1) [34]. Four models were fit: (1) A null model with only neighborhood perceptions and block-group random effect; (2–3) two partial models with individual, objective, and perceived neighborhood characteristics; and (4) a full model adjusting for all covariates described in the previous section. Akaike information criterion (AIC) values were used to evaluate improvement between models, and *p*-values were calculated based on Satterthwaite’s approximations [35].

For Analysis II (variables related to the accuracy of participants’ perceptions regarding perceived supermarket access), we compared covariates of participants who accurately reported their access to those of overestimators, under-estimators, and participants who reported not knowing the distance to the nearest supermarket) [36]. A series of four multivariate log-linear models were fit via neural networks (using the multinom function of the nnet package in R): (1) A null model that only included neighborhood perceptions, (2–3) two partial models with individual and objective neighborhood covariates, and (4) a full model adjusting for all potential covariates described above [37,38]. AIC values were calculated to assess improvement between the null and full models. Likelihood ratio tests were used to assess overall variation between accuracy categories within covariates, and z-tests were used identify significant covariates within categories [39]. Output tables were generated using the sjPlot and stargazer R packages [40,41]. 

## 3. Results

### 3.1. Study Population

Most participants reported that the nearest supermarket was within a 5, 10, or 20-min walk (47.0%), while 40.0% perceived living further than a 20-min walk, and 13.2% provided a “Don’t Know” response. Averages and counts of sociodemographic and built environment variables by distance perception category are fully reported in Table 1 and were aggregated for ease of presentation.

### 3.2. Analysis I: Associations with Objective Supermarket Distance

**Figure 1:**
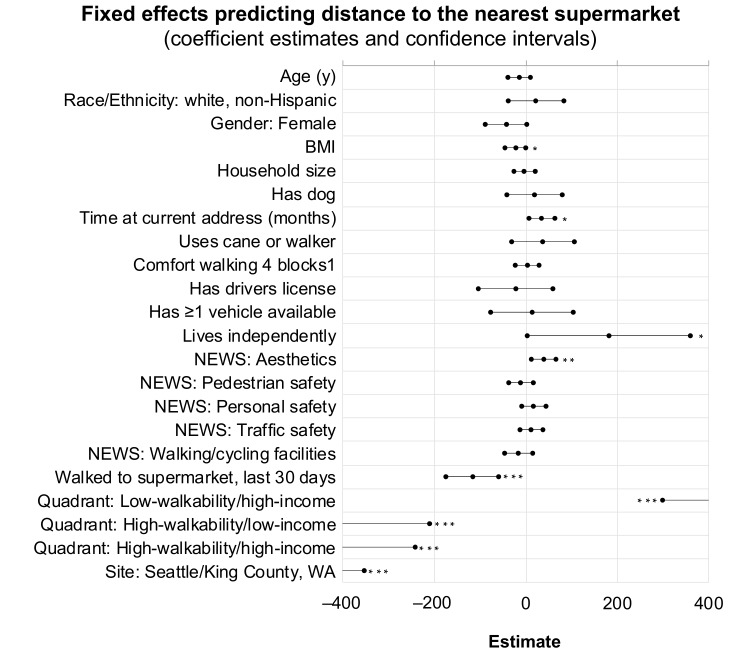
Coefficient estimates and confidence intervals for fixed effects predicting an individual’s actual distance to nearest grocery store. Note: Significance levels indicated by: *** *p* < 0.001; ** *p* < 0.01; * *p* < 0.05.

Participant characteristics, perceptions, and behaviors were significantly related to increasing objective supermarket distance: Lower BMI (β = −22.89, *p* = 0.048), more favorable self-reported neighborhood aesthetics (β = 39.02, *p* = 0.005), longer time lived at the current address (β = 35.29, *p* = 0.015), living independently (β = 181.49, *p* = 0.046), and having walked to the nearest supermarket within the last 30 days (β = −117.14, *p* < 0.001). Additionally, the quadrant in which a participant resided was significantly related to objective distance to a supermarket (further distance in the low-walkability/high-income quadrant and shorter distance in the high-walkability/low-income and high-walkability/high-income quadrants, compared to the low-walkability, low-income quadrant) and their residence in Seattle/King County, WA versus Baltimore/Washington, DC was negatively related to objective distance (all *p* < 0.001). Table 2 shows the results of the final model, and Figure 1 represents variable coefficients and confidence intervals in a forest plot. 

### 3.3. Analysis II: Accuracy of Perceived Supermarket Access in Relation to Objective Access

**Figure 2:**
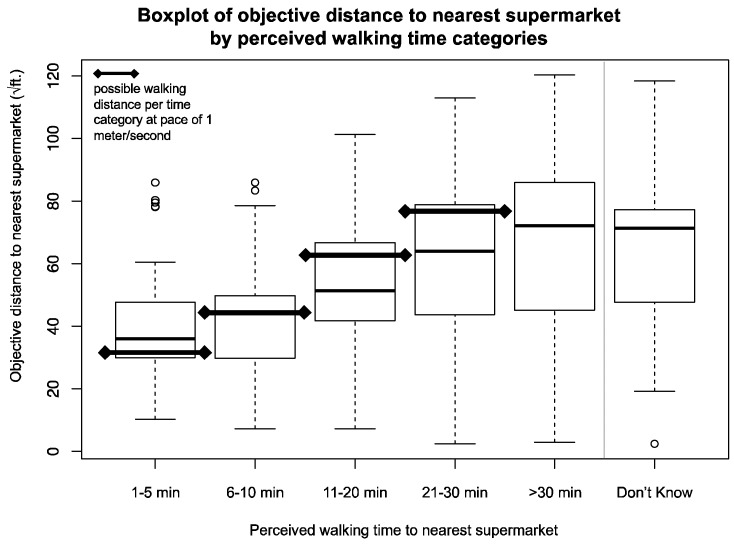
Boxplot of objective distance to the nearest grocery store and perceived walking time. Note: Distance presented as square root for ease of presentation, and lines with markers demarcate the possible distances traveled by walking for a given time at pace of 1 m/s.

Following the investigation of influential individual and neighborhood factors correlated with actual distance to the nearest supermarket, we investigated further the putative reasons underlying individuals’ misperceptions of supermarket access. Assuming (based on previous literature [31]) that a speed of one meter per second is reasonably representative of an older adult’s walking pace, we compared perceived walk time with objectively-measured distance between participants’ homes and the nearest supermarket. As Figure 2 shows, the majority of the study sample misjudged their physical food access by either underestimating the distance (e.g., perceived living closer to a supermarket than they actually did; 33.9%), overestimating (e.g., perceived living further from a supermarket than they actually did; 24.0%) or responding “Don’t Know” (13.2%). Only 29.0 percent of participants accurately reported the walking time to the nearest supermarket (*n* = 255). Table 3 provides a summary of significant differences in sociodemographic characteristics by these accuracy classifications. Output tables for partial models from Analyses I and II are available as Supplemental Material (Supplemental Tables S1 and S2, respectively).

**Figure 3:**
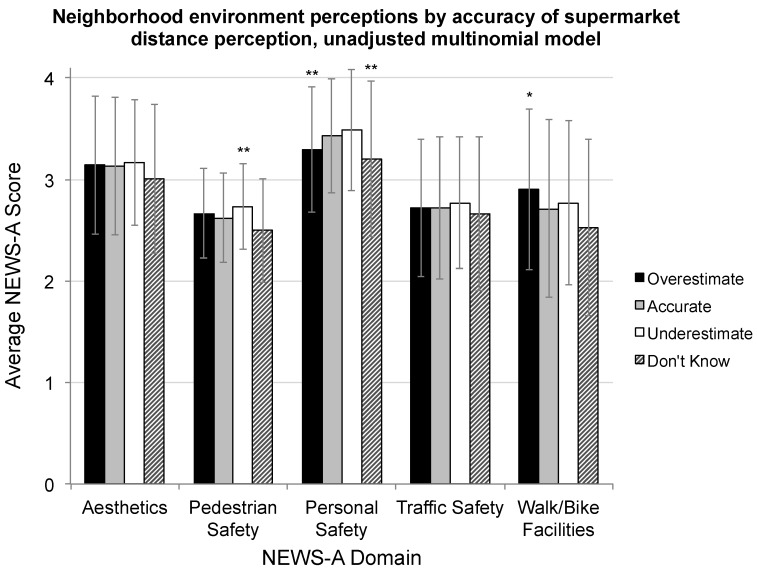
Average accurate, over-, and underestimator perceptions of neighborhood environment measured with the abbreviated NEWS (NEWS-A) instrument. Note: ** *p* < 0.01; * *p* < 0.05 for z-test of coefficients for distance perception categories compared to accurate in unadjusted multinomial log-linear model.

**Figure 4:**
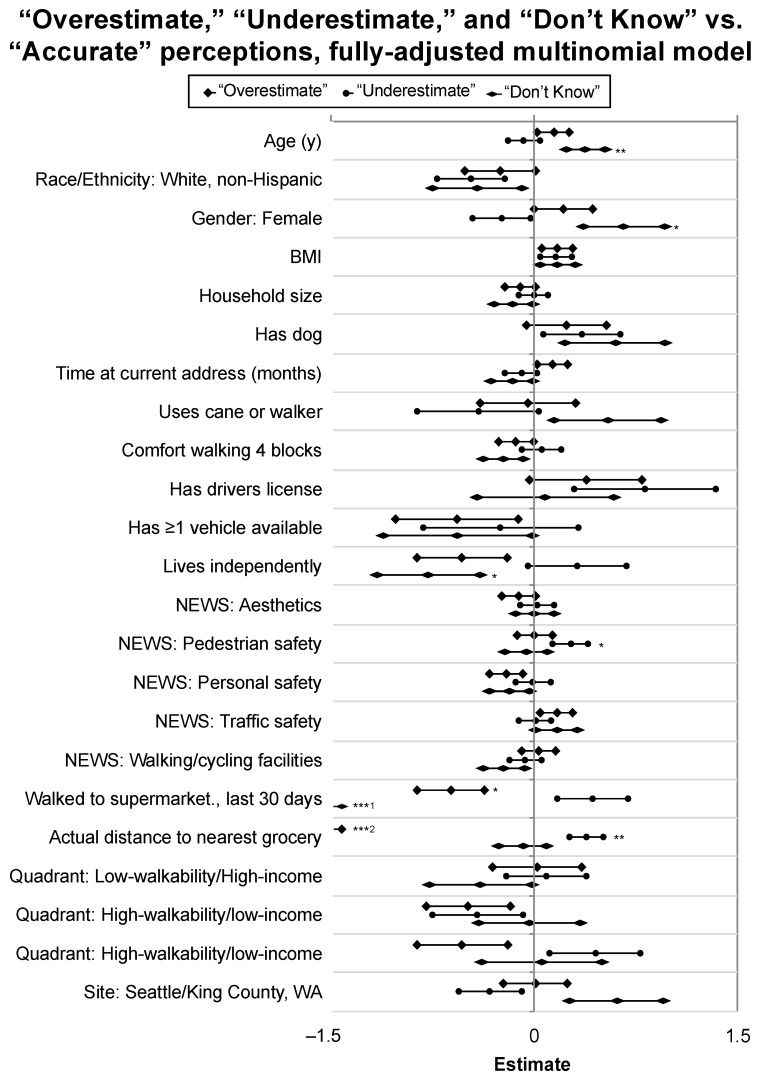
Multinomial log-linear model results: Predictor coefficient estimates and standard errors for overestimators, underestimators, and individuals reporting “Don’t Know” for perceived walking distance to the closest grocery store, compared to individuals with accurate perceptions. Note: Significance levels (determined by z-test) indicated by: *** *p* < 0.001; ** *p* < 0.01; * *p* < 0.05. ^1^ Actual coefficient estimate not shown: −15.48 (standard error< 0.001). ^2^ Actual coefficient estimate not shown: −1.92 (standard error = 0.18).

The unadjusted model comparing the accuracy of perception to NEWS-A measures found significant relationships between inaccurate distance perceptions and perceived personal safety: Lower personal safety scores were associated with both overestimated and “Don’t Know” responses. A higher perceived availability of walking and bicycling facilities was associated with overestimating distance, and underestimation was associated with higher perceived pedestrian safety (see Figure 3). In the fully-adjusted model, compared to participants with accurate perceptions of supermarket access, an “Underestimator” status was significantly and positively related to perceived neighborhood pedestrian safety (*p* = 0.04) and distance to the nearest retailer (*p* = 0.003) (see Figure 4). Compared to the “Accurate” group, an “Overestimator” status was significantly and negatively related to having walked to the supermarket in the last 30 days (*p* = 0.01), and negatively related to objective distance (*p* < 0.001), compared to the accurate group. Finally, compared to the accurate group, a “Don’t Know” status was significantly and positively associated with age (*p* = 0.01) and identifying as a woman (*p* = 0.03), and negatively associated with living independently (*p* = 0.04). Likelihood ratio tests for coefficients in the fully-adjusted multinomial model are reported in Table 3. A complete summary of coefficients, confidence intervals, and *p*-values is provided in Supplemental Table S2. 

Sensitivity analyses using assumed walking speeds of 0.8 and 1.2 m/s (slightly slower/faster than the 1.0 m/s speed used here) are summarized in Supplemental Material Table S3 Overall, while some covariates changed in their level of significance under slower/faster speed assumptions, none changed in terms of the direction of association (e.g., positive versus negative relationships). Notably, however, one of the largest changes in magnitude of association was in the areas of individual mobility for “Underestimators” versus “Accurate” participants, especially those who used a cane or walker: −1.19, (95% CI −1.99, −0.39) under a 0.8 m/s assumption, and −0.41 (95% CI −1.29, 0.46) under the 1.0 m/s assumption. The second sensitivity analysis (performed with a dataset that excluded participants with possible mobility constraints) yielded similar results, though with reduced statistical significance for all of the NEWS neighborhood perception variables (see Supplemental Material Table S4). These findings raise important considerations that are further elaborated in the Limitations. 

## 4. Discussion

Although 29.0% of participants had accurate estimates of their proximity to a supermarket, a large remainder either over- or underestimated their level of physical access to a supermarket: 24.0% were underestimators, or had “optimistic” feelings about their neighborhood food access (e.g., they perceived living closer to a supermarket than they actually did), while 33.9% were overestimators, or had “pessimistic” feelings about their neighborhood food access (e.g., they perceived living further from a supermarket than they actually did). These groups were characterized by both perceived and objective neighborhood factors. Compared to accurate participants, underestimators reported significantly higher feelings of pedestrian safety in their neighborhoods, while overestimators were significantly less likely to have walked to the supermarket in the last 30 days. In terms of the objective food environment, overestimators actually lived significantly closer to supermarkets than more accurate participants, while underestimators lived significantly farther away, consistent with other studies of perceived distance to neighborhood amenities among children and adults [42]. Of note, approximately 13.2% of participants could not identify the distance to the closest supermarket; compared to the “Accurate” participants, this group was significantly older, more likely to be women than men, and not living independently. Additionally, “Don’t Know” respondents were significantly less likely to have walked to the supermarket in the last 30 days. 

Several findings provide insights into how the relationship between the objective and perceived food environments may be moderated. First, travel on foot to the supermarket (e.g., having a relatively recent experience walking to the supermarket) was strongly and positively related to the ability to provide an accurate estimate (compared to responding “Don’t Know”), and not having recently walked to the supermarket was also a significant predictor of perceiving the distance to be much greater than it was. Second, individual factors, such as gender (“Don’t Know” respondents were more likely to be women than accurate respondents), age (“Don’t Know” respondents were significantly older), and living independently (“Don’t Know” respondents were less likely to live independently) were related to the accuracy of perceptions. Finally, neighborhood perceptions, such as higher perceived pedestrian safety, were associated with more “optimistic” perceptions of the (shorter) time required to walk compared to those with accurate perceptions. 

The results of the linear mixed-model examining correlates of objective distance to the nearest supermarket help to place these findings in a broader context. Only neighborhood aesthetics were significantly related to objective supermarket distance, with higher scores correlated with greater objective distance from a supermarket. While in the current sample, aesthetics did not appear to play a role in helping to define an individual’s sense of distance, perception of pedestrian safety was significantly and positively related to underestimating distance.

Some of the influential environmental factors associated with older adults’ food environment perceptions may be modifiable (e.g., walking safety programs may improve perceptions of pedestrian safety; installation of new or improved walking/cycling facilities may raise awareness of these resources), though other significant variables in this study, such as an individual’s place of residence or ability to live independently, are much less so. If modifiable perceived or objective environmental factors are related to older adults’ reports of more “optimistic” or “pessimistic” views of their food environments, future interventions could seek to encourage seniors to more fully engage with the neighborhood food environment available to them. This finding aligns with the conclusions of Park and colleagues, who suggest that individual values and interests are important and measurable factors for behavioral interventions (which, in their study, concerned meeting physical activity recommendations), and with the earlier work of Thorndyke and Hayes-Roth, which compared the effects of actual experiences versus a map study in terms of distance estimation [43]. Specific to the food environment, relevant interventions that employ direct resident engagement in conducting assessments of neighborhood resources, including different kinds of food retailers, may help to set the stage for modifying individual perceptions of the food environment [44,45,46]. In turn, lowered perceived barriers about accessing food environment resources, such as walking distance to a retailer or neighborhood safety, could lead to opportunities for other diet-related interventions that rely on changing food shopping behaviors. Additionally, this study highlights specific individual factors, like age and gender, which should be further investigated in terms of their effects on the accuracy of distance perceptions, which could be important in designing future interventions in this area.

### Limitations

This study has several limitations. First, as a secondary analysis of a cross-sectional observational study, we cannot establish causal relationships between variables and the primary outcomes. Second, we focused on supermarkets as the largest and most identifiable type of food retailer to ensure better agreement between participants’ perceptions and what was objectively measured, though the food environment includes many other potential sources, such as fruit and vegetable markets and convenience stores and restaurants. Additionally, our translation of walking minutes to distance was based on averages, which will have varying degrees of error for specific individuals. 

Individual-level variables, such as physical ability, may also influence both the perceived environment, including distance to neighborhood resources, and one’s walking speed, potentially introducing risk of misclassifying participants as over- or underestimators based on the average speed. Our sensitivity analyses offer some contextual detail on this point and underscore the interrelated nature of individual characteristics and neighborhood perceptions. Different methodological approaches, such as structural equation modeling, could provide possible future avenues to disentangle these effects, particularly in studies with prospective, as opposed to cross-sectional, designs. 

Finally, other qualitative and quantitative studies have shown how shoppers select food retailers according to a variety of individual and store-level criteria beyond physical distance to one’s home. While we had rich individual-level data, we were not able to include information related to store features, such as pricing, quality, or cultural tailoring, which have been found to be influential elsewhere [47,48,49], nor were we able to test whether or not participants were aware of the “objectively” nearest store.

## 5. Conclusions

In this study, we assessed individual and neighborhood-level correlates of older adults’ perceptions of living close to a supermarket across a population that lived in two U.S. regions with varying levels of walkability and income. We also tested these perceptions against objective GIS-based measures of the food environment, and classified participants as accurate, or over- or underestimators. Our results reveal how perceptions of over- or underestimating one’s level of access to a supermarket include individual characteristics and capabilities, as well as neighborhood pedestrian safety perceptions. Further research is needed to understand how these food environment perceptions form, how they are associated with actual food choice, and whether or not they can be modified by interventions in order to promote positive health behavior outcomes.

## Figures and Tables

**Table 1:** Individual and neighborhood characteristics of participant population, by perceived walking time to closest supermarket retailer. NEWS: Neighborhood environment walkability survey, BMI: Body mass index.

	<20 min Walk	≥20 min Walk	Don’t Know
**Counts (%)**	(n = 413)	(n = 351)	(n = 116)
Gender			
Male	206 (0.50)	152 (0.43)	27 (0.23)
Female	207 (0.50)	199 (0.57)	89 (0.77)
Race			
Not white	119 (0.29)	101 (0.29)	42 (0.37)
White (not Hispanic)	292 (0.71)	249 (0.71)	73 (0.63)
Lives independently			
Yes	361 (0.87)	286 (0.81)	69 (0.59)
No	52 (0.13)	65 (0.19)	47 (0.41)
Has a valid driver’s license			
Yes	368 (0.89)	310 (0.89)	91 (0.78)
No	45 (0.11)	40 (0.11)	25 (0.22)
Uses a cane or walker			
Yes	35 (0.08)	37 (0.11)	34 (0.29)
No	377 (0.92)	314 (0.89)	82 (0.71)
Quadrant			
1. Low-Walkability/Low-Income	62 (0.15)	89 (0.25)	32 (0.28)
2. Low-Walkability/High-Income	67 (0.16)	128 (0.36)	32 (0.28)
3. High-Walkability/Low-Income	137 (0.33)	83 (0.24)	36 (0.31)
4. High-Walkability/High-Income	147 (0.36)	51 (0.15)	16 (0.14)
Site			
Baltimore/Washington, DC	198 (0.48)	172 (0.49)	57 (0.49)
Seattle/King County, WA	215 (0.52)	179 (0.51)	59 (0.51)
≥1 vehicle available			
Yes	366 (0.89)	310 (0.89)	88 (0.76)
No	47 (0.11)	40 (0.11)	28 (0.24)
Dog owner			
Yes	57 (0.14)	48 (0.14)	18 (0.16)
No	356 (0.86)	302 (0.86)	98 (0.84)
Walked to supermarket in last 30 days			
Yes	227 (0.55)	307 (0.87)	116 (1.00)
No	185 (0.45)	44 (0.13)	0 (0.00)
**Mean (SD)**			
Age (y)	74.74 (6.78)	75.17 (6.5)	78.02 (7.28)
BMI	26.11 (4.31)	26.5 (4.95)	27.6 (5.87)
Household size (persons)	1.75 (0.68)	1.74 (0.79)	1.47 (0.75)
Can walk 4 blocks (scale 1–10)	8.84 (2.53)	7.93 (3.22)	6.26 (3.9)
Time at current address (months)	249.57 (189.81)	274.53 (189.84)	209.16 (191.4)
NEWS Aesthetic score	3.19 (0.63)	3.1 (0.7)	3 (0.73)
NEWS Traffic safety score	2.76 (0.66)	2.71 (0.7)	2.66 (0.76)
NEWS Pedestrian safety score	2.73 (0.42)	2.6 (0.45)	2.5 (0.51)
NEWS Personal safety score	3.42 (0.56)	3.36 (0.64)	3.2 (0.77)
NEWS Walk/cycle facilities score	2.89 (0.75)	2.7 (0.9)	2.53 (0.87)
Objective distance to supermarket (ft)	2696 (1947)	4719 (3019)	4630 (2724)

**Table 2:** Fully-adjusted mixed model with block group random effect: Individual and neighborhood correlates of actual distance to nearest grocery store.

	Outcome: Actual Distance to Grocery Store (in Meters)		
*Predictors*	*Beta*	*CI*	*p*
(Intercept)	1416.86	1180.01–1653.72	<0.001
Age (y)	−14.45	−39.04–10.14	0.250
Race/Ethnicity: white, non-Hispanic	22.42	−38.37–83.21	0.470
Gender: Female	−43.29	−88.77–2.20	0.063
BMI	−22.89	−45.56–−0.23	0.048
Household size	−2.67	−25.94–20.60	0.822
Has dog	19.13	−41.45–79.72	0.536
Time at current address (months)	35.29	6.97–63.60	0.015
Uses cane or walker	37.68	−30.85–106.22	0.282
Comfort walking 4 blocks^1^	2.95	−23.08–28.99	0.824
Has driver’s license	−22.26	−103.82–59.31	0.593
Has ≥ 1 vehicle available	13.41	−76.92–103.74	0.771
Lives independently	181.49	3.12–359.87	0.046
NEWS: Aesthetics	39.02	12.14–65.89	0.005
NEWS: Pedestrian Safety	−10.52	−37.42–16.38	0.444
NEWS: Personal Safety	17.57	−9.00–44.15	0.195
NEWS: Traffic Safety	12.29	−12.79–37.36	0.337
NEWS: Walking/Cycling Facilities	−15.68	−46.40–15.05	0.318
Walked to nearest grocery, last 30 days	−117.14	−174.86–−59.42	<0.001
Quadrant: Low-Walk/High-Inc^2^	534.04	299.05–769.02	<0.001
Quadrant: High-Walk/Low-Inc^2^	−409.30	−608.16–−210.45	<0.001
Quadrant: High-Walk/High-Inc^2^	−489.56	−737.33–−241.78	<0.001
Site: Seattle/King County, WA^3^	−530.46	−707.69–−353.22	<0.001
Observations	868		
R^2^/Ω_0_^2^	0.926/0.925		
AIC	12600.926		

^1^ Comfort walking 4 blocks (10-pt Likert scale); ^2^ Compared to Quadrant 1 (Low-Walkability, Low-Income); ^3^ Compared to Baltimore/Washington DC. AIC: Akaike information criterion.

**Table 3:** Likelihood ratio tests for coefficients in the fully-adjusted multinomial model comparing participants categorized by accuracy of perceptions regarding distance to nearest supermarket.

	Likelihood Ratio Chi-Square	*p*-Value
Age (y)	9.74	0.02 *
Race/Ethnicity: White, non-Hispanic	4.01	0.26
Gender: Female	8.91	0.03 *
BMI	3.33	0.34
Household size	1.78	0.62
Has dog	3.21	0.36
Time at current address (months)	5.09	0.17
Uses cane or walker	4.52	0.21
Comfort walking 4 blocks	3.72	0.29
Has driver license	3.03	0.39
Has ≥1 vehicle available	1.86	0.60
Lives independently	8.58	0.04 *
NEWS: Aesthetics	1.21	0.75
NEWS: Pedestrian Safety	5.75	0.12
NEWS: Personal Safety	3.68	0.30
NEWS: Traffic Safety	2.74	0.43
NEWS: Walking/Cycling Facilities	3.04	0.39
Walked to nearest grocery, last 30 days	58.86	<0.001 ***
Actual distance to nearest grocery	216.13	<0.001 ***
Quadrant	16.86	0.05
Site: Seattle/King County, WA	6.87	0.08

Note: *** *p* < 0.001; * *p* < 0.05.

## Supplementary Materials

The following are available online at http://www.mdpi.com/2308-3417/4/1/11/s1, Tables S1: Output from null, partial, and full linear mixed models predicting objective distance to the nearest supermarket with block group random effect, Tables S2: Output from null, partial, and full multivariate log-linear models predicting likelihood of over-estimating, under-estimating, or responding “don’t know”, compared to accurately estimating the distance to the nearest supermarket, Tables S3: Participant counts of distance perception accuracy categories by assumed walking speed, Tables S4: Sensitivity analysis using reduced dataset that excludes participants with possible mobility constraints.
