# Supplementary material for: How Well Do Seniors Estimate Distance to Food? The Accuracy of Older Adults’ Reported Proximity to Local Grocery Stores
Source: Geriatrics (Basel). 2019 Jan 10;4(1):11. doi: 10.3390/geriatrics4010011 (PMC6473472; doi:10.3390/geriatrics4010011)
Supplement: Supplementary file 1 [file geriatrics-04-00011-s001.zip › TABLE S1.docx]

**Supplemental Table S1**: Output from null, partial, and full linear mixed models predicting objective distance to the nearest supermarket with block group random effect

|  |  | Null Model | | |  | Partial Model 1 | | |  | Partial Model 2 | | |  | Partial Model 3 | | |  | Full Model | | |
| --- | --- | --- | --- | --- | --- | --- | --- | --- | --- | --- | --- | --- | --- | --- | --- | --- | --- | --- | --- | --- |
|  |  | B | CI | p |  | B | CI | p |  | B | CI | p |  | B | CI | p |  | B | CI | p |
| Fixed Parts | | | | | | | | | | | | | | | | | | | | |
| (Intercept) |  | 1178.36 | 1074.56 – 1282.15 | **<.001** |  | 1121.98 | 985.57 – 1258.39 | **<.001** |  | 1126.29 | 990.07 – 1262.51 | **<.001** |  | 1116.00 | 981.84 – 1250.17 | **<.001** |  | 1106.16 | 985.19 – 1227.13 | **<.001** |
| Age (yrs) |  | -5.27 | -28.52 – 17.98 | .657 |  | -8.56 | -33.09 – 15.98 | .494 |  | -8.82 | -33.37 – 15.73 | .482 |  | -15.41 | -39.99 – 9.16 | .219 |  | -14.45 | -39.04 – 10.14 | .250 |
| Race/Ethnicity: white, non-Hispanic |  | -9.93 | -40.27 – 20.42 | .522 |  | -4.46 | -35.02 – 26.09 | .775 |  | -6.77 | -37.28 – 23.73 | .664 |  | -8.38 | -38.69 – 21.92 | .588 |  | -11.21 | -41.60 – 19.18 | .470 |
| Gender: female |  | 15.70 | -6.15 – 37.55 | .160 |  | 13.97 | -8.73 – 36.67 | .228 |  | 19.67 | -3.21 – 42.55 | .093 |  | 21.45 | -1.30 – 44.20 | .065 |  | 21.64 | -1.10 – 44.39 | .063 |
| BMI |  | -19.14 | -40.76 – 2.47 | .083 |  | -17.71 | -40.43 – 5.00 | .127 |  | -16.84 | -39.53 – 5.85 | .146 |  | -21.97 | -44.64 – 0.70 | .058 |  | -22.89 | -45.56 – -0.23 | **.048** |
| Household size |  | 4.39 | -18.58 – 27.35 | .708 |  | 0.07 | -23.37 – 23.52 | .995 |  | -3.83 | -27.26 – 19.59 | .748 |  | -2.09 | -25.38 – 21.20 | .860 |  | -2.67 | -25.94 – 20.60 | .822 |
| Has dog |  |  |  |  |  | -10.80 | -41.41 – 19.82 | .490 |  | -8.35 | -38.83 – 22.13 | .592 |  | -11.16 | -41.47 – 19.15 | .471 |  | -9.57 | -39.86 – 20.73 | .536 |
| Time at current address (months) |  |  |  |  |  | 43.58 | 14.87 – 72.29 | **.003** |  | 41.41 | 12.80 – 70.02 | **.005** |  | 39.57 | 11.14 – 68.00 | **.007** |  | 35.29 | 6.97 – 63.60 | **.015** |
| Uses cane or walker |  |  |  |  |  | -17.35 | -51.77 – 17.07 | .324 |  | -18.83 | -53.24 – 15.58 | .284 |  | -18.35 | -52.54 – 15.85 | .293 |  | -18.84 | -53.11 – 15.42 | .282 |
| Comfort walking 4 blocks |  |  |  |  |  | -2.50 | -28.14 – 23.14 | .848 |  | -5.86 | -31.89 – 20.18 | .659 |  | 0.72 | -25.32 – 26.77 | .957 |  | 2.95 | -23.08 – 28.99 | .824 |
| Has drivers license |  |  |  |  |  | 5.60 | -35.47 – 46.68 | .789 |  | 7.87 | -33.10 – 48.83 | .707 |  | 9.88 | -30.84 – 50.60 | .635 |  | 11.13 | -29.65 – 51.91 | .593 |
| Has >=1 vehicle available |  |  |  |  |  | -21.10 | -66.49 – 24.29 | .363 |  | -15.66 | -60.85 – 29.54 | .497 |  | -5.70 | -50.85 – 39.44 | .805 |  | -6.70 | -51.87 – 38.46 | .771 |
| Lives independently |  |  |  |  |  | -67.99 | -159.64 – 23.65 | .146 |  | -74.10 | -165.58 – 17.39 | .113 |  | -60.89 | -151.74 – 29.96 | .189 |  | -90.75 | -179.93 – -1.56 | **.046** |
| NEWS: Aesthetics |  |  |  |  |  |  |  |  |  | 39.63 | 12.58 – 66.68 | **.004** |  | 39.15 | 12.28 – 66.02 | **.004** |  | 39.02 | 12.14 – 65.89 | **.005** |
| NEWS: Pedestrian Safety |  |  |  |  |  |  |  |  |  | -14.79 | -41.69 – 12.11 | .281 |  | -8.30 | -35.20 – 18.60 | .546 |  | -10.52 | -37.42 – 16.38 | .444 |
| NEWS: Personal Safety |  |  |  |  |  |  |  |  |  | 20.33 | -6.30 – 46.96 | .135 |  | 19.89 | -6.57 – 46.34 | .141 |  | 17.57 | -9.00 – 44.15 | .195 |
| NEWS: Traffic Safety |  |  |  |  |  |  |  |  |  | 12.06 | -13.18 – 37.31 | .349 |  | 10.94 | -14.15 – 36.03 | .393 |  | 12.29 | -12.79 – 37.36 | .337 |
| NEWS: Walking/Cycling Facilities |  |  |  |  |  |  |  |  |  | -14.20 | -45.23 – 16.84 | .370 |  | -17.04 | -47.88 – 13.80 | .279 |  | -15.68 | -46.40 – 15.05 | .318 |
| Has ever walked to nearest grocery |  |  |  |  |  |  |  |  |  |  |  |  |  | 63.21 | 34.38 – 92.05 | **<.001** |  | 58.57 | 29.71 – 87.43 | **<.001** |
| Actual distance to the nearest grocery |  |  |  |  |  |  |  |  |  |  |  |  |  |  |  |  |  | 91.21 | -42.35 – 224.76 | .182 |
| Quadrant: Low-Walk/High-Inc |  |  |  |  |  |  |  |  |  |  |  |  |  |  |  |  |  | 625.24 | 472.23 – 778.25 | **<.001** |
| Quadrant: High-Walk/Low-Inc |  |  |  |  |  |  |  |  |  |  |  |  |  |  |  |  |  | -318.10 | -450.10 – -186.10 | **<.001** |
| Quadrant: High-Walk/High-Inc |  |  |  |  |  |  |  |  |  |  |  |  |  |  |  |  |  | 265.23 | 176.61 – 353.85 | **<.001** |
| Random Parts | | | | | | | | | | | | | | | | | | | | |
| σ^2^ |  | 71494.015 | | |  | 70884.860 | | |  | 69919.995 | | |  | 69085.130 | | |  | 69686.978 | | |
| τ_00, blkgrp_ |  | 592958.845 | | |  | 589980.529 | | |  | 590284.700 | | |  | 564385.451 | | |  | 377545.807 | | |
| N_blkgrp_ |  | 227 | | |  | 227 | | |  | 227 | | |  | 227 | | |  | 227 | | |
| ICC_blkgrp_ |  | 0.892 | | |  | 0.893 | | |  | 0.894 | | |  | 0.891 | | |  | 0.844 | | |
| Observations |  | 869 | | |  | 868 | | |  | 868 | | |  | 868 | | |  | 868 | | |
| R^2^ / Ω_0_^2^ |  | .923 / .922 | | |  | .924 / .924 | | |  | .926 / .925 | | |  | .927 / .926 | | |  | .926 / .925 | | |
| AIC |  | 12861.651 | | |  | 12793.321 | | |  | 12754.538 | | |  | 12731.119 | | |  | 12616.175 | | |
