# Supplementary material for: How Well Do Seniors Estimate Distance to Food? The Accuracy of Older Adults’ Reported Proximity to Local Grocery Stores
Source: Geriatrics (Basel). 2019 Jan 10;4(1):11. doi: 10.3390/geriatrics4010011 (PMC6473472; doi:10.3390/geriatrics4010011)
Supplement: Supplementary file 1 [file geriatrics-04-00011-s001.zip › Table S3.docx]

**Supplemental Material Table S3**

Participant counts of distance perception accuracy categories by assumed walking speed

|  | Accurate | Over-Estimate | Under-Estimate | Don’t Know |
| --- | --- | --- | --- | --- |
| 0.8 meters/second | 263 | 226 | 275 | 116 |
| 1.0 meters/second | 255 | 298 | 211 | 116 |
| 1.2 meters/second | 244 | 389 | 131 | 116 |

| Sensitivity analysis: Output from null, partial, and full multivariate log-linear models predicting likelihood of over-estimating, under-estimating, or responding “don’t know”, compared to accurately estimating the distance to the nearest supermarket (0.8 m/s, 1.0 m/s, 1.2 m/s walking speed assumptions) | | | | | | | | | | | | | | | | | | |
| --- | --- | --- | --- | --- | --- | --- | --- | --- | --- | --- | --- | --- | --- | --- | --- | --- | --- | --- |
|  | | | | | | | | | | | | | | | | | | |
|  | *Dependent variable: Type of distance perception (over-estimate, under-estimate, don’t know; reference group: accurate)* | | | | | | | | | | | | | | | | | |
|  |  | | | | | | | | | | | | | | | | | |
|  | Individual and Socio-Demographic Covariates | | | | | | | | | All Covariates | | | | | | | | |
|  | 0.8 m/s | | | 1.0 m/s | | | 1.2 m/s | | | 0.8 m/s | | | 1.0 m/s | | | 1.2 m/s | | |
|  | 2.overestimate | 3.underestimate | 4.dontknow | 2.overestimate | 3.underestimate | 4.dontknow | 2.overestimate | 3.underestimate | 4.dontknow | 2.overestimate | 3.underestimate | 4.dontknow | 2.overestimate | 3.underestimate | 4.dontknow | 2.overestimate | 3.underestimate | 4.dontknow |
|  | (1) | (2) | (3) | (4) | (5) | (6) | (7) | (8) | (9) | (10) | (11) | (12) | (13) | (14) | (15) | (16) | (17) | (18) |
|  | | | | | | | | | | | | | | | | | | |
| Age (yrs) | 0.27 (0.08, 0.45) | -0.33 (-0.52, -0.13) | 0.48 (0.24, 0.71) | 0.30 (0.12, 0.48) | -0.20 (-0.41, 0.01) | 0.57 (0.33, 0.82) | 0.29 (0.11, 0.46) | -0.16 (-0.41, 0.09) | 0.62 (0.37, 0.86) | 0.10 (-0.16, 0.35) | -0.25 (-0.46, -0.03) | 0.30 (0.02, 0.58) | 0.14 (-0.08, 0.37) | -0.08 (-0.31, 0.16) | 0.38 (0.09, 0.66) | 0.12 (-0.09, 0.34) | -0.10 (-0.38, 0.18) | 0.37 (0.08, 0.66) |
|  | p = 0.01^***^ | p = 0.001^***^ | p = 0.0001^***^ | p = 0.002^***^ | p = 0.07^*^ | p = 0.0000^***^ | p = 0.002^***^ | p = 0.21 | p = 0.0000^***^ | p = 0.45 | p = 0.03^**^ | p = 0.04^**^ | p = 0.22 | p = 0.53 | p = 0.01^***^ | p = 0.27 | p = 0.49 | p = 0.02^**^ |
| Race/Ethnicity: white, non-Hispanic | 0.08 (-0.35, 0.51) | -0.19 (-0.57, 0.20) | -0.41 (-0.92, 0.10) | -0.16 (-0.56, 0.24) | -0.39 (-0.80, 0.03) | -0.55 (-1.06, -0.03) | 0.02 (-0.36, 0.40) | -0.45 (-0.91, 0.01) | -0.44 (-0.96, 0.08) | 0.03 (-0.56, 0.61) | -0.22 (-0.67, 0.24) | -0.29 (-0.93, 0.34) | -0.25 (-0.75, 0.26) | -0.47 (-0.96, 0.03) | -0.42 (-1.06, 0.23) | 0.05 (-0.42, 0.52) | -0.47 (-1.04, 0.09) | -0.27 (-0.92, 0.38) |
|  | p = 0.72 | p = 0.34 | p = 0.12 | p = 0.43 | p = 0.07^*^ | p = 0.04^**^ | p = 0.92 | p = 0.06^*^ | p = 0.10^*^ | p = 0.93 | p = 0.35 | p = 0.37 | p = 0.34 | p = 0.07^*^ | p = 0.21 | p = 0.83 | p = 0.10^*^ | p = 0.42 |
| Gender: female | 0.10 (-0.29, 0.48) | -0.33 (-0.69, 0.03) | 0.84 (0.31, 1.37) | 0.22 (-0.15, 0.58) | -0.30 (-0.69, 0.08) | 0.93 (0.40, 1.47) | 0.19 (-0.15, 0.54) | -0.17 (-0.62, 0.27) | 0.99 (0.45, 1.53) | 0.18 (-0.31, 0.67) | -0.26 (-0.65, 0.13) | 0.61 (0.02, 1.20) | 0.22 (-0.21, 0.64) | -0.24 (-0.66, 0.18) | 0.66 (0.07, 1.25) | 0.13 (-0.27, 0.53) | -0.13 (-0.63, 0.36) | 0.68 (0.09, 1.28) |
|  | p = 0.63 | p = 0.08^*^ | p = 0.002^***^ | p = 0.25 | p = 0.13 | p = 0.001^***^ | p = 0.27 | p = 0.45 | p = 0.0004^***^ | p = 0.48 | p = 0.20 | p = 0.05^**^ | p = 0.33 | p = 0.27 | p = 0.03^**^ | p = 0.52 | p = 0.60 | p = 0.03^**^ |
| BMI | 0.12 (-0.07, 0.32) | 0.06 (-0.12, 0.25) | 0.36 (0.14, 0.58) | 0.19 (0.01, 0.38) | 0.11 (-0.10, 0.31) | 0.42 (0.19, 0.64) | 0.12 (-0.06, 0.30) | 0.13 (-0.10, 0.36) | 0.39 (0.16, 0.61) | 0.10 (-0.15, 0.35) | 0.08 (-0.13, 0.29) | 0.12 (-0.13, 0.37) | 0.17 (-0.05, 0.39) | 0.16 (-0.07, 0.39) | 0.17 (-0.08, 0.43) | 0.04 (-0.17, 0.25) | 0.11 (-0.16, 0.37) | 0.10 (-0.15, 0.36) |
|  | p = 0.21 | p = 0.51 | p = 0.002^***^ | p = 0.05^**^ | p = 0.31 | p = 0.0003^***^ | p = 0.20 | p = 0.28 | p = 0.001^***^ | p = 0.45 | p = 0.45 | p = 0.34 | p = 0.14 | p = 0.18 | p = 0.18 | p = 0.73 | p = 0.43 | p = 0.42 |
| Household size | -0.23 (-0.43, -0.03) | -0.02 (-0.20, 0.15) | -0.28 (-0.54, -0.02) | -0.18 (-0.37, 0.003) | 0.03 (-0.15, 0.22) | -0.27 (-0.53, -0.002) | -0.16 (-0.34, 0.01) | -0.01 (-0.23, 0.20) | -0.29 (-0.55, -0.02) | -0.14 (-0.39, 0.11) | -0.07 (-0.26, 0.13) | -0.16 (-0.45, 0.12) | -0.10 (-0.32, 0.12) | -0.003 (-0.21, 0.21) | -0.15 (-0.44, 0.13) | -0.06 (-0.26, 0.14) | -0.03 (-0.28, 0.21) | -0.15 (-0.44, 0.14) |
|  | p = 0.03^**^ | p = 0.81 | p = 0.04^**^ | p = 0.06^*^ | p = 0.73 | p = 0.05^**^ | p = 0.07^*^ | p = 0.90 | p = 0.04^**^ | p = 0.27 | p = 0.51 | p = 0.26 | p = 0.36 | p = 0.98 | p = 0.29 | p = 0.58 | p = 0.80 | p = 0.30 |
| Has dog |  |  |  |  |  |  |  |  |  | 0.27 (-0.39, 0.93) | 0.25 (-0.28, 0.77) | 0.55 (-0.17, 1.26) | 0.24 (-0.35, 0.82) | 0.35 (-0.20, 0.91) | 0.60 (-0.13, 1.32) | 0.08 (-0.47, 0.62) | 0.49 (-0.14, 1.12) | 0.56 (-0.17, 1.28) |
|  |  |  |  |  |  |  |  |  |  | p = 0.43 | p = 0.36 | p = 0.14 | p = 0.43 | p = 0.22 | p = 0.11 | p = 0.79 | p = 0.13 | p = 0.14 |
| Time at current address (months) |  |  |  |  |  |  |  |  |  | 0.19 (-0.05, 0.44) | -0.01 (-0.22, 0.20) | -0.15 (-0.45, 0.14) | 0.13 (-0.09, 0.35) | -0.10 (-0.32, 0.13) | -0.16 (-0.46, 0.14) | 0.06 (-0.14, 0.27) | 0.02 (-0.25, 0.30) | -0.15 (-0.45, 0.16) |
|  |  |  |  |  |  |  |  |  |  | p = 0.13 | p = 0.90 | p = 0.32 | p = 0.24 | p = 0.42 | p = 0.29 | p = 0.55 | p = 0.87 | p = 0.35 |
| Uses cane or walker |  |  |  |  |  |  |  |  |  | -0.71 (-1.45, 0.03) | -1.19 (-1.99, -0.39) | 0.11 (-0.63, 0.86) | -0.04 (-0.73, 0.65) | -0.41 (-1.29, 0.46) | 0.54 (-0.23, 1.31) | 0.47 (-0.23, 1.18) | -0.003 (-1.07, 1.07) | 0.95 (0.13, 1.77) |
|  |  |  |  |  |  |  |  |  |  | p = 0.06^*^ | p = 0.004^***^ | p = 0.77 | p = 0.91 | p = 0.36 | p = 0.17 | p = 0.19 | p = 1.00 | p = 0.03^**^ |
| Comfort walking 4 blocks |  |  |  |  |  |  |  |  |  | -0.28 (-0.56, 0.004) | -0.13 (-0.38, 0.12) | -0.30 (-0.58, -0.01) | -0.13 (-0.38, 0.12) | 0.06 (-0.22, 0.34) | -0.23 (-0.51, 0.06) | -0.04 (-0.28, 0.21) | 0.04 (-0.28, 0.36) | -0.19 (-0.48, 0.10) |
|  |  |  |  |  |  |  |  |  |  | p = 0.06^*^ | p = 0.32 | p = 0.04^**^ | p = 0.31 | p = 0.70 | p = 0.12 | p = 0.78 | p = 0.81 | p = 0.20 |
| Has drivers license |  |  |  |  |  |  |  |  |  | 0.55 (-0.32, 1.42) | 0.63 (-0.24, 1.50) | 0.11 (-0.86, 1.07) | 0.38 (-0.43, 1.19) | 0.82 (-0.20, 1.84) | 0.08 (-0.90, 1.07) | 0.09 (-0.71, 0.89) | 0.94 (-0.35, 2.24) | -0.12 (-1.12, 0.89) |
|  |  |  |  |  |  |  |  |  |  | p = 0.22 | p = 0.16 | p = 0.83 | p = 0.36 | p = 0.12 | p = 0.87 | p = 0.83 | p = 0.16 | p = 0.83 |
| Has >=1 vehicle available |  |  |  |  |  |  |  |  |  | -0.57 (-1.50, 0.37) | -0.31 (-1.27, 0.64) | -0.51 (-1.56, 0.53) | -0.57 (-1.45, 0.32) | -0.25 (-1.37, 0.87) | -0.56 (-1.63, 0.51) | 0.02 (-0.85, 0.89) | -0.41 (-1.74, 0.93) | -0.22 (-1.32, 0.88) |
|  |  |  |  |  |  |  |  |  |  | p = 0.24 | p = 0.52 | p = 0.34 | p = 0.21 | p = 0.67 | p = 0.31 | p = 0.97 | p = 0.56 | p = 0.70 |
| Lives independently |  |  |  |  |  |  |  |  |  | -0.46 (-1.20, 0.28) | 0.18 (-0.46, 0.82) | -0.80 (-1.53, -0.06) | -0.53 (-1.18, 0.12) | 0.32 (-0.40, 1.03) | -0.78 (-1.53, -0.03) | -0.78 (-1.41, -0.14) | -0.17 (-1.03, 0.68) | -1.10 (-1.88, -0.32) |
|  |  |  |  |  |  |  |  |  |  | p = 0.23 | p = 0.59 | p = 0.04^**^ | p = 0.11 | p = 0.39 | p = 0.05^**^ | p = 0.02^**^ | p = 0.70 | p = 0.01^***^ |
| NEWS: Aesthetics |  |  |  |  |  |  |  |  |  | -0.31 (-0.60, -0.02) | -0.13 (-0.35, 0.09) | -0.06 (-0.34, 0.22) | -0.11 (-0.35, 0.13) | 0.03 (-0.22, 0.27) | 0.003 (-0.28, 0.28) | 0.09 (-0.13, 0.31) | 0.23 (-0.05, 0.51) | 0.10 (-0.18, 0.38) |
|  |  |  |  |  |  |  |  |  |  | p = 0.04^**^ | p = 0.26 | p = 0.67 | p = 0.37 | p = 0.84 | p = 0.99 | p = 0.44 | p = 0.12 | p = 0.48 |
| NEWS: Pedestrian Safety |  |  |  |  |  |  |  |  |  | 0.05 (-0.25, 0.34) | 0.16 (-0.07, 0.39) | -0.07 (-0.38, 0.23) | 0.003 (-0.26, 0.26) | 0.27 (0.01, 0.52) | -0.06 (-0.37, 0.25) | 0.19 (-0.05, 0.43) | 0.30 (0.01, 0.59) | 0.02 (-0.29, 0.34) |
|  |  |  |  |  |  |  |  |  |  | p = 0.75 | p = 0.18 | p = 0.65 | p = 0.99 | p = 0.04^**^ | p = 0.70 | p = 0.13 | p = 0.05^**^ | p = 0.88 |
| NEWS: Personal Safety |  |  |  |  |  |  |  |  |  | -0.07 (-0.34, 0.21) | 0.15 (-0.08, 0.38) | -0.08 (-0.37, 0.21) | -0.21 (-0.45, 0.03) | -0.01 (-0.26, 0.25) | -0.18 (-0.48, 0.11) | -0.14 (-0.37, 0.09) | -0.02 (-0.31, 0.27) | -0.18 (-0.48, 0.12) |
|  |  |  |  |  |  |  |  |  |  | p = 0.64 | p = 0.21 | p = 0.60 | p = 0.10^*^ | p = 0.96 | p = 0.23 | p = 0.24 | p = 0.90 | p = 0.25 |
| NEWS: Traffic Safety |  |  |  |  |  |  |  |  |  | 0.01 (-0.26, 0.28) | -0.13 (-0.35, 0.08) | 0.07 (-0.23, 0.37) | 0.17 (-0.07, 0.40) | 0.01 (-0.22, 0.24) | 0.17 (-0.14, 0.47) | -0.07 (-0.29, 0.14) | -0.09 (-0.36, 0.19) | 0.05 (-0.25, 0.36) |
|  |  |  |  |  |  |  |  |  |  | p = 0.97 | p = 0.24 | p = 0.64 | p = 0.16 | p = 0.95 | p = 0.29 | p = 0.51 | p = 0.54 | p = 0.74 |
| NEWS: Walking/Cycling Facilities |  |  |  |  |  |  |  |  |  | 0.23 (-0.07, 0.53) | -0.01 (-0.22, 0.21) | -0.17 (-0.47, 0.12) | 0.04 (-0.21, 0.29) | -0.07 (-0.30, 0.17) | -0.22 (-0.52, 0.07) | 0.02 (-0.21, 0.24) | 0.03 (-0.24, 0.30) | -0.22 (-0.52, 0.08) |
|  |  |  |  |  |  |  |  |  |  | p = 0.13 | p = 0.95 | p = 0.25 | p = 0.78 | p = 0.59 | p = 0.15 | p = 0.90 | p = 0.84 | p = 0.16 |
| Walked to nearest grocery, last 30 days |  |  |  |  |  |  |  |  |  | -0.87 (-1.42, -0.33) | 0.19 (-0.29, 0.66) | -16.24 (-16.24, -16.24) | -0.61 (-1.10, -0.13) | 0.43 (-0.08, 0.95) | -15.48 (-15.48, -15.48) | -0.78 (-1.24, -0.31) | 0.24 (-0.38, 0.86) | -16.07 (-16.07, -16.07) |
|  |  |  |  |  |  |  |  |  |  | p = 0.002^***^ | p = 0.45 | p = 0.00^***^ | p = 0.02^**^ | p = 0.10^*^ | p = 0.00^***^ | p = 0.001^***^ | p = 0.45 | p = 0.00^***^ |
| Objective distance to grocery store |  |  |  |  |  |  |  |  |  | -3.22 (-3.79, -2.65) | 0.20 (-0.05, 0.44) | -0.06 (-0.41, 0.28) | -1.92 (-2.29, -1.56) | 0.38 (0.13, 0.64) | -0.08 (-0.43, 0.26) | -1.42 (-1.72, -1.12) | 0.55 (0.26, 0.83) | -0.12 (-0.47, 0.22) |
|  |  |  |  |  |  |  |  |  |  | p = 0.00^***^ | p = 0.12 | p = 0.72 | p = 0.00^***^ | p = 0.003^***^ | p = 0.63 | p = 0.00^***^ | p = 0.0003^***^ | p = 0.49 |
| Quadrant: Low-Walk/High-Inc |  |  |  |  |  |  |  |  |  | 1.05 (0.25, 1.85) | 0.45 (-0.09, 0.99) | -0.10 (-0.84, 0.64) | 0.02 (-0.62, 0.66) | 0.09 (-0.49, 0.67) | -0.39 (-1.13, 0.34) | -0.42 (-1.01, 0.17) | -0.34 (-1.00, 0.32) | -0.69 (-1.44, 0.06) |
|  |  |  |  |  |  |  |  |  |  | p = 0.01^***^ | p = 0.11 | p = 0.80 | p = 0.95 | p = 0.77 | p = 0.30 | p = 0.17 | p = 0.32 | p = 0.08^*^ |
| Quadrant: High-Walk/Low-Inc |  |  |  |  |  |  |  |  |  | -0.40 (-1.11, 0.31) | -0.13 (-0.71, 0.44) | 0.14 (-0.57, 0.85) | -0.49 (-1.10, 0.12) | -0.42 (-1.07, 0.24) | -0.03 (-0.76, 0.70) | -0.44 (-1.03, 0.15) | -0.74 (-1.53, 0.05) | -0.11 (-0.86, 0.64) |
|  |  |  |  |  |  |  |  |  |  | p = 0.28 | p = 0.65 | p = 0.71 | p = 0.12 | p = 0.22 | p = 0.94 | p = 0.15 | p = 0.07^*^ | p = 0.78 |
| Quadrant: High-Walk/High-Inc |  |  |  |  |  |  |  |  |  | -0.25 (-1.00, 0.51) | 0.63 (0.02, 1.23) | 0.36 (-0.50, 1.22) | -0.53 (-1.18, 0.13) | 0.45 (-0.20, 1.11) | 0.06 (-0.81, 0.93) | -0.93 (-1.55, -0.31) | -0.36 (-1.14, 0.41) | -0.38 (-1.26, 0.50) |
|  |  |  |  |  |  |  |  |  |  | p = 0.53 | p = 0.05^**^ | p = 0.42 | p = 0.12 | p = 0.18 | p = 0.90 | p = 0.004^***^ | p = 0.36 | p = 0.41 |
| Site: Seattle/King County |  |  |  |  |  |  |  |  |  | 0.10 (-0.42, 0.62) | -0.26 (-0.69, 0.18) | 0.62 (-0.05, 1.29) | 0.01 (-0.45, 0.47) | -0.32 (-0.79, 0.14) | 0.61 (-0.07, 1.28) | -0.05 (-0.48, 0.37) | -0.26 (-0.81, 0.30) | 0.56 (-0.12, 1.24) |
|  |  |  |  |  |  |  |  |  |  | p = 0.71 | p = 0.25 | p = 0.07^*^ | p = 0.97 | p = 0.17 | p = 0.08^*^ | p = 0.81 | p = 0.37 | p = 0.11 |
| Constant | -0.29 (-0.72, 0.15) | 0.30 (-0.09, 0.68) | -1.22 (-1.79, -0.66) | 0.16 (-0.24, 0.57) | 0.20 (-0.21, 0.61) | -1.13 (-1.70, -0.56) | 0.38 (-0.002, 0.76) | -0.26 (-0.72, 0.20) | -1.18 (-1.75, -0.61) | -1.14 (-2.42, 0.14) | -0.34 (-1.41, 0.73) | -0.43 (-1.66, 0.81) | 0.70 (-0.35, 1.75) | -0.80 (-2.04, 0.45) | -0.15 (-1.41, 1.12) | 1.39 (0.38, 2.41) | -0.59 (-2.07, 0.89) | 0.22 (-1.08, 1.51) |
|  | p = 0.20 | p = 0.14 | p = 0.0001^***^ | p = 0.44 | p = 0.35 | p = 0.0002^***^ | p = 0.06^*^ | p = 0.28 | p = 0.0001^***^ | p = 0.08^*^ | p = 0.54 | p = 0.50 | p = 0.20 | p = 0.22 | p = 0.83 | p = 0.01^***^ | p = 0.44 | p = 0.75 |
|  | | | | | | | | | | | | | | | | | | |
| Akaike Inf. Crit. | 2,242.55 | 2,242.55 | 2,242.55 | 2,238.93 | 2,238.93 | 2,238.93 | 2,137.23 | 2,137.23 | 2,137.23 | 1,843.50 | 1,843.50 | 1,843.50 | 1,912.77 | 1,912.77 | 1,912.77 | 1,852.32 | 1,852.32 | 1,852.32 |
|  | | | | | | | | | | | | | | | | | | |
| *Note:* | ^*^p<0.1; ^**^p<0.05; ^***^p<0.01 | | | | | | | | | | | | | | | | | |
