# Supplementary material for: How Well Do Seniors Estimate Distance to Food? The Accuracy of Older Adults’ Reported Proximity to Local Grocery Stores
Source: Geriatrics (Basel). 2019 Jan 10;4(1):11. doi: 10.3390/geriatrics4010011 (PMC6473472; doi:10.3390/geriatrics4010011)
Supplement: Supplementary file 1 [file geriatrics-04-00011-s001.zip › Table S4.docx]

**Supplemental Material Table S4:** Sensitivity analysis using reduced dataset that excludes participants with possible mobility constraints

| Type of Distance Perception | Count (% total) |
| --- | --- |
| Accurate | 185 (31.7) |
| Over-estimate | 183 (31.4) |
| Under-estimate | 170 (29.2) |
| Don’t know | 45 (7.7) |

Output from full multivariate log-linear models predicting likelihood of over-estimating, under-estimating, or responding “don’t know”, compared to accurately estimating the distance to the nearest supermarket (1.0 m/s walking speed assumption)

|  |  |  |  |
| --- | --- | --- | --- |
|  | *Dependent variable: Type of distance perception*  *(over-estimate, under-estimate, don’t know; reference group: accurate)* | | |
|  | 2.overestimate | 3.underestimate | 4.dontknow |
|  | (13) | (14) | (15) |
| Age (yrs) | 0.14 (-0.11, 0.40) | -0.20 (-0.45, 0.06) | 0.45 (0.09, 0.80) |
|  | p = 0.27 | p = 0.13 | p = 0.02^**^ |
| Race/Ethnicity: white, non-Hispanic | -0.43 (-1.05, 0.18) | -0.56 (-1.13, 0.01) | -0.37 (-1.26, 0.52) |
|  | p = 0.17 | p = 0.06^*^ | p = 0.42 |
| Gender: female | 0.15 (-0.36, 0.66) | -0.42 (-0.90, 0.06) | 0.72 (-0.08, 1.51) |
|  | p = 0.57 | p = 0.09^*^ | p = 0.08^*^ |
| BMI | 0.005 (-0.25, 0.26) | 0.13 (-0.12, 0.38) | 0.22 (-0.13, 0.56) |
|  | p = 0.98 | p = 0.32 | p = 0.23 |
| Household size | 0.01 (-0.26, 0.28) | 0.12 (-0.12, 0.36) | -0.16 (-0.54, 0.22) |
|  | p = 0.96 | p = 0.33 | p = 0.40 |
| Has dog | 0.08 (-0.58, 0.74) | 0.37 (-0.24, 0.98) | 0.52 (-0.38, 1.42) |
|  | p = 0.82 | p = 0.24 | p = 0.27 |
| Time at current address (months) | 0.17 (-0.08, 0.41) | -0.06 (-0.31, 0.19) | -0.25 (-0.61, 0.11) |
|  | p = 0.19 | p = 0.66 | p = 0.18 |
| Has drivers license | 0.97 (-0.26, 2.21) | 1.19 (-0.23, 2.62) | -0.50 (-2.06, 1.06) |
|  | p = 0.13 | p = 0.10^*^ | p = 0.53 |
| Has >=1 vehicle available | -0.99 (-2.37, 0.40) | -0.77 (-2.39, 0.84) | -0.53 (-2.57, 1.52) |
|  | p = 0.17 | p = 0.35 | p = 0.62 |
| NEWS: Aesthetics | -0.12 (-0.41, 0.18) | 0.19 (-0.09, 0.47) | 0.19 (-0.18, 0.57) |
|  | p = 0.44 | p = 0.18 | p = 0.31 |
| NEWS: Pedestrian Safety | 0.05 (-0.28, 0.37) | 0.26 (-0.04, 0.56) | -0.22 (-0.67, 0.24) |
|  | p = 0.79 | p = 0.09^*^ | p = 0.36 |
| NEWS: Personal Safety | -0.10 (-0.39, 0.18) | -0.04 (-0.32, 0.24) | 0.07 (-0.36, 0.50) |
|  | p = 0.49 | p = 0.80 | p = 0.77 |
| NEWS: Traffic Safety | -0.04 (-0.33, 0.25) | -0.07 (-0.33, 0.20) | -0.03 (-0.46, 0.40) |
|  | p = 0.78 | p = 0.64 | p = 0.89 |
| NEWS: Walking/Cycling Facilities | 0.16 (-0.14, 0.46) | -0.11 (-0.39, 0.16) | -0.16 (-0.58, 0.27) |
|  | p = 0.30 | p = 0.42 | p = 0.48 |
| Walked to nearest grocery, last 30 days | -0.90 (-1.48, -0.31) | 0.41 (-0.18, 1.00) | -15.74 (-15.74, -15.74) |
|  | p = 0.003^***^ | p = 0.18 | p = 0.00^***^ |
| Objective distance to grocery store | -2.16 (-2.67, -1.66) | 0.39 (0.09, 0.68) | -0.02 (-0.50, 0.45) |
|  | p = 0.00^***^ | p = 0.02^**^ | p = 0.92 |
| Quadrant: Low-Walk/High-Inc | 0.01 (-0.76, 0.78) | 0.14 (-0.53, 0.81) | -0.24 (-1.26, 0.77) |
|  | p = 0.99 | p = 0.69 | p = 0.65 |
| Quadrant: High-Walk/Low-Inc | -1.00 (-1.76, -0.24) | -0.49 (-1.27, 0.28) | 0.24 (-0.80, 1.29) |
|  | p = 0.02^**^ | p = 0.21 | p = 0.66 |
| Quadrant: High-Walk/High-Inc | -0.54 (-1.34, 0.26) | 0.73 (-0.02, 1.48) | 0.40 (-0.79, 1.59) |
|  | p = 0.19 | p = 0.06^*^ | p = 0.51 |
| Site: Seattle/King County | 0.27 (-0.27, 0.80) | -0.23 (-0.75, 0.30) | 0.84 (-0.04, 1.73) |
|  | p = 0.33 | p = 0.40 | p = 0.07^*^ |
| Constant | 0.06 (-1.33, 1.44) | -0.30 (-1.93, 1.32) | -1.08 (-3.11, 0.95) |
|  | p = 0.94 | p = 0.72 | p = 0.30 |
